# Individualized Decision Making in Transperineal Prostate Biopsy: Should All Men Undergo an Additional Systematic Biopsy?

**DOI:** 10.3390/cancers14215230

**Published:** 2022-10-25

**Authors:** August Sigle, Rodrigo Suarez-Ibarrola, Matthias Benndorf, Moritz Weishaar, Jonathan Morlock, Arkadiusz Miernik, Christian Gratzke, Cordula A. Jilg, Markus Grabbert

**Affiliations:** 1 Department of Urology, Faculty of Medicine, Medical Center—University of Freiburg, 79106 Freiburg, Germany; 2 Berta-Ottenstein-Programme, Faculty of Medicine, University of Freiburg, 79110 Freiburg, Germany; 3 Department of Radiology, Faculty of Medicine, Medical Center—University of Freiburg, 79106 Freiburg, Germany

**Keywords:** prostatic neoplasms, image-guided biopsy, fusion biopsy, biopsy strategy

## Abstract

**Simple Summary:**

In the last few years, multiparametric magnetic resonance imaging (mpMRI) has been implemented in the diagnostic prostate cancer pathway for the identification of cancerous lesions, and consecutively, targeted fusion biopsy was implemented. In some cases, aggressive prostate cancer is missed by a targeted biopsy. To address this imperfection, additional systematic biopsy is recommended but may be harmful in terms of the additional diagnosis of indolent cancer, and the higher frequency of adverse events and resource expenditures. This study investigates whether all men should undergo an additional systematic biopsy within this clinically relevant trade-off. As a key finding, men with an mpMRI-lesion classified as PI-RADS 5 may obviate additional systematic biopsy. This was confirmed when we analyzed histopathological reclassification rates between biopsy and a subsequent radical prostatectomy.

**Abstract:**

Background: In prostate cancer (PC) diagnosis, additional systematic biopsy (SB) is recommended to complement MRI-targeted biopsy (TB) to address the limited sensitivity of TB alone. The combination of TB+SB is beneficial for diagnosing additional significant PC (sPC) but harmful in terms of the additional diagnosis of indolent PC (iPC), morbidity, and resource expenditures. We aimed to investigate the benefit of additional SB and to identify predictors for this outcome. Methods: We analyzed the frequency of upgrading to sPC by additional SB in a retrospective single-center cohort of 1043 men. Regression analysis (RA) was performed to identify predictors for this outcome. Reclassification rates of ISUP grade groups between prostate biopsy and a subsequent radical prostatectomy were assessed. Results: Additional SB led to upgrading to sPC in 98/1043 men (9.4%) and to the additional diagnosis of iPC in 71/1043 (6.8%). In RA, men harboring a PI-RADS 2-4 lesion were more likely to have TB results upgraded by SB (*p* < 0.01) compared to PI-RADS 5 men. When analyzing reclassification rates, additional SB reduced the upgrading to sPC from 43/214 (20.1%) to 8/214 (3.7%). In the PI-RADS 5 subgroup, this difference decreased: 4/87 (4.7%) with TB only vs. 1/87 (1.2%) with TB+SB. Conclusion: Men with a PI-RADS 5 lesion may obviate additional SB.

## 1. Introduction 

The significant value of MRI-targeted biopsy (TB) compared to systematic biopsy (SB) alone for the detection of clinically significant prostate cancer (sPC) has been confirmed in recent prospective clinical trials such as PROMIS [1], PRECISION [2], and MRI-FIRST [3]. 

However, despite the advantages of TB, such as reducing overdiagnosis of indolent cancers (iPC), morbidity, operative time, and pathologists’ workload, there is a concern over the unacceptable proportion of missed high-grade cancers when SB is omitted [3]. Among the main shortcomings of TB are (1) Reading errors: Misdiagnosing lesions due to misinterpretation; (2) Presence of non-MRI visible sPC; and (3) Targeting errors by the person performing the TB [4]. A recent meta-analysis evaluating cancer detection rates (CDR) for TB versus SB found that omitting SB would miss approximately 16% of sPC [5]. 

Since systematic cores increase the detection of iPC [5], morbidity [6], and resource expenditures, a compromise needs to be reached to limit SB in men that are more likely to benefit from it. 

There is growing interest in determining if a subgroup of men might benefit from SB in addition to TB. Previous studies have identified the clinical setting (biopsy naïve, previous negative biopsy, active surveillance), age, prostate volume, MRI-lesion volume and the PI-RADS Score [7,8,9] predictive for upgrading to sPC by SB. A recent study proposed patients’ PI-RADS score as a promising tool to select the optimal biopsy strategy [10]. However, these findings are limited to a cohort who underwent transrectal prostate biopsy. Current guidelines favor the transperineal approach for prostate biopsy [11]. Moreover, the evaluation of reclassification rates of prostate cancer (PC) grade groups between prostate biopsy and a subsequent radical prostatectomy (RP) are missing. We aimed (1) to evaluate the frequency of upgrading to sPC by SB over TB and to identify predictors for this outcome; (2) to analyze CDRs by TB and TB+SB for transperineal prostate biopsy stratified by the identified parameters; and (3) to investigate reclassification rates of PC grade groups between prostate biopsy and a subsequent RP stratified by the identified parameters.

## 2. Patients and Methods 

### 2.1. Study Population

We analyzed a retrospective single-center cohort of 1043 men (Total Cohort) who underwent prostate biopsy. The indication for biopsy was based on suspicious prostate-specific antigen (PSA) levels/dynamics, abnormal digital rectal examination, or as a part of an active surveillance routine. Men with very high PSA-levels (>20 ng/mL) and suspicion of locally advanced disease were included in this study.

Men who underwent a prostate biopsy between October 2015–May 2020 were included in the study. Men were excluded if only TB or only SB was conducted, or in the case of incomplete clinical data. Men who were diagnosed with PC and underwent RP within one year after biopsy were considered for further analysis (Prostatectomy Cohort). Data collection was approved by the local Ethics Committee (ETK 21-1191). The study was performed in accordance with the Declaration of Helsinki.

### 2.2. MR Imaging, Biopsy Procedure and Histopathological Analysis

All men had a pre-biopsy multiparametric magnetic resonance imaging (mpMRI) according to the current Prostate Imaging Reporting and Data System (PI-RADS) [12]. Image interpretation was performed by a group of board-certified radiologists as a part of the clinical routine and without central revision. Robot-assisted mpMRI/transrectal ultrasound fusion biopsy of the prostate (iSRobot Mona LisaTM^®^, Biobot Surgical, Singapore) was performed as a combined procedure of TB plus synchronous SB. The Ginsburg protocol that addresses both sides of the prostate was applied for SB planning [13]. The median total number of cores taken was 35 (interquartile range (IQR) 31–40) with a median of 31 (IQR 26–34) systematic biopsy cores.

SB was not performed blinded to MRI lesions but was planned independently from TB. Eight different surgeons performed the standardized biopsy procedures that were included in this study. Procedural details were described previously [14]. Prostate biopsy was performed in lithotomy position via the transperineal route and under general anaesthesia. Antibiotic prophylaxis and local anaesthesia were not administered.

All biopsy cores were labelled, processed and analysed individually by a group of board-certified uropathologists and according to the International Society of Urological Pathology (ISUP) standards [15].

### 2.3. Data collection and Statistical Analysis

Demographic and clinical data were extracted by reviewing patients’ electronic medical records.

Baseline characteristics included age, previous biopsy and active surveillance status, PSA, prostate volume by MRI, mpMRI findings according to PI-RADS, zonal and side-specific information on target localization, index lesion volume, number of lesions, the number of biopsy cores and histopathological findings from prostate biopsy according to ISUP. The index lesion was defined as the lesion with the highest PI-RADS grade and in case of several equally assessed lesions the one with the largest volume was considered.

Continuous variables were described as median with interquartile range (IQR). Categorical variables were described with integers and percentages. 

The primary endpoint of the study was the upgrading to sPC by additional SB. Clinically significant prostate cancer was defined as the presence of any PC classified as ISUP grade group 2 or higher. As secondary endpoints, we aimed to identify predictors for upgrading to sPC, and we assessed reclassification rates of ISUP grade groups between prostate biopsy and a subsequent RP. To identify predictors for the primary endpoint, we performed binary logistic regression analysis, including the following covariates: age, previous biopsy and active surveillance status, PSA, prostate volume, PI-RADS score, zonal and side-specific target localization, index lesion volume, number of lesions and the number of TB and SB cores. For the selection of variables in the multivariable analysis, we applied backward stepwise elimination. The binary cut-off for index lesion volume was set at 0.6 mL with respect to the variable’s median. The McNemar’s test was used to compare the detection rates of sPC. Moreover, we analyzed the overlap of 95% confidence intervals (CI) of the respective detection rates. *p*-value < 0.05 was considered statistically significant. SPSS© software (SPSS statistics 27) was used for statistical analysis.

## 3. Results 

### 3.1. Study Cohort

**Figure 1:**
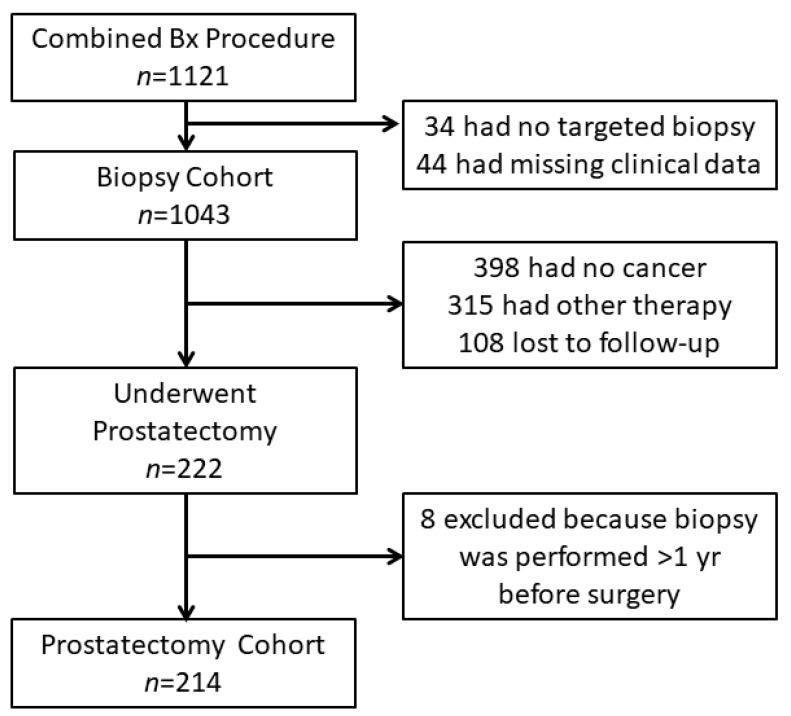
Enrollment and Outcomes. All included men underwent combined biopsy (Bx) procedure with a combination of MRI-targeted biopsy plus synchronous systematic biopsy. For men diagnosed with PC different treatment options were offered (active surveillance, radiotherapy, and radical prostatectomy). Men undergoing a subsequent radical prostatectomy were considered for the analysis of reclassification rates of cancer grade groups between biopsy and wholemount specimen.

The enrollment and outcomes are presented in Figure 1. A total of 1121 men underwent prostate biopsy at the University Hospital Freiburg, Germany between October 2015–May 2020. Thirty-four men had no TB and 44 were excluded due to missing clinical data, resulting in a total cohort of 1043 men. Among these men, 222 patients (21.3%) underwent subsequent RP. Eight men of this subgroup were excluded because the time between biopsy and surgery exceeded one year, resulting in a prostatectomy cohort of 214 men.

### 3.2. Baseline Characteristics 

Baseline characteristics and CDRs are illustrated in Table 1. Median age and PSA were 67.0 years (interquartile range (IQR) 61.0–72.0) and 8.8 ng/mL (6.0–12.6), respectively. In the prostatectomy cohort, the median age and PSA were 67.0 years and (62.0–72.0) and 9.3 ng/mL (6.3–14.0), respectively. 

### 3.3. Cancer Detection Rates and Upgrading in Systematic Versus MRI–Targeted Biopsy

In the total cohort 649/1043 (62.2%) men were diagnosed with PC, with 521/1043 (50.0%) men harboring sPC. The CDRs of any PC and sPC by TB/SB were 48.7%/59.5% and 40.6%/46.3%, respectively.

Additional SB led to an upgrading to sPC in 98/1043 men (9.4%). Out of these men, 70/98 (71.4%) had no PC detected by TB and 28/98 (28.6%) were upgraded from iPC. Systematic biopsy led to the additional diagnosis of iPC in 71/1043 (6.8%) men.

### 3.4. Regression Analysis for the Upgrading to sPC by Systematic Biopsy

To identify predictors for upgrading to sPC by SB, we calculated both univariate and multivariate regression analysis (Table 2). In univariate regression analysis, we found the PI-RADS category to be significantly associated with the study’s primary endpoint: men harboring a PI-RADS lesion 2–4 were more likely to have TB results upgraded by SB (*p* < 0.01) compared to men with a PI-RADS 5 lesion. This finding was consistent in multivariable analysis. Moreover, we found patients with non-peripheral zone lesions being less likely to be upgraded by SB (OR 0.44 (0.23–0.85; *p* < 0.01)) compared to men with lesions localized in the peripheral zone. Smaller index lesions with volumes < 0.6 mL were associated with higher rates of upgrading to sPC by SB (OR 2.15 (CI 1.38–3.34; *p* < 0.01). In addition, we found a significant association of prostate volume and the event of upgrading to sPC by SB (OR 0.99 (0.98–1.00, *p* < 0.03) in multivariate analysis.

### 3.5. Cancer Detection Rates Stratified by PI-RADS Score

**Figure 2:**
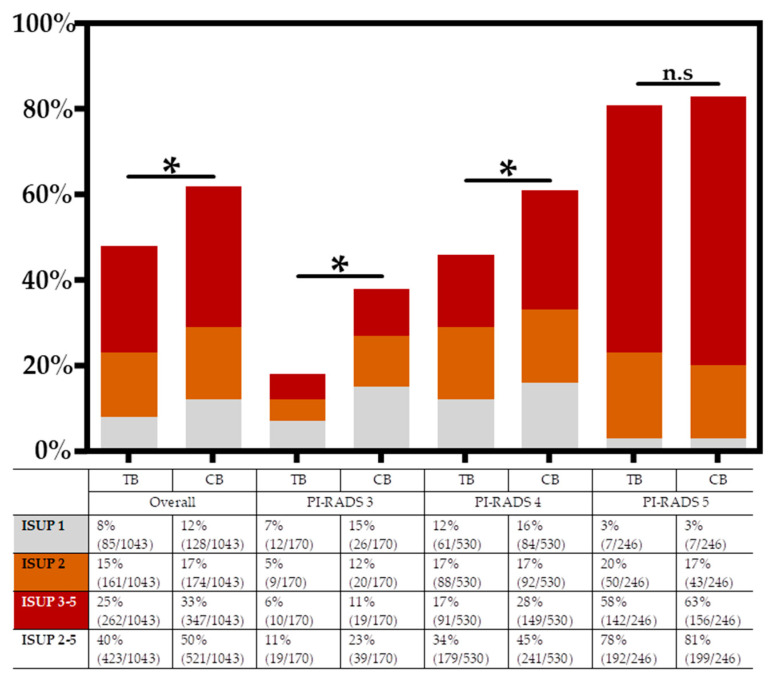
Cancer Detection Rates stratified by PI-RADS score and biopsy method. Additional systematic biopsy diagnosed significantly (*) more clinically significant prostate cancer (sPC) in the total cohort and in the subgroups of PI-RADS 2–4 men. For the group of men with a PI-RADS 5 lesion this difference was not statistically significant (n.s) when considering the overlap of 95% confidence intervals: sPC by TB was 78.0% (73.0–83.0%) vs. 80.9% (75.9–85.8%) for CB. TB—MRI-targeted biopsy; CB—combined biopsy = TB plus synchronous systematic biopsy.

After the identification of the PI-RADS Score as a main influencing factor for the effect of SB, we stratified CDRs for TB vs. TB+SB by PI-RADS groups. The results are illustrated in Figure 2. With respect to the total cohort, the combined biopsy strategy (CB) diagnosed significantly more sPC and iPC compared to TB only (50.0% vs. 40.6%; *p* < 0.001 and 6.8% vs 2.9%, *p* < 0.001). This effect was consistent for the subgroup of men with PI-RADS 3 and PI-RADS 4 lesions, with an upgrading to sPC by SB in 20/170 (11.8%) and 62/530 (11.7%), respectively. When comparing the detection rates of sPC for CB versus TB in men classified as PI-RADS 5, there was no significant difference considering the overlap of 95% CIs: 78.0% (73.0–83.0%) for TB vs. 80.9% (75.9–85.8%) for CB. Omitting SB in men with a PI-RADS 5 lesion would have missed the diagnosis of any PC in 7/1043 (0.7%) men, with 1/1043 (0.1%) classified as ISUP 1, 3/1043 (0.3%) ISUP 2 and 3/1043 (0.3%) ISUP 3–5.

### 3.6. Reclassification in Subsequent Radical Prostatectomy

**Figure 3:**
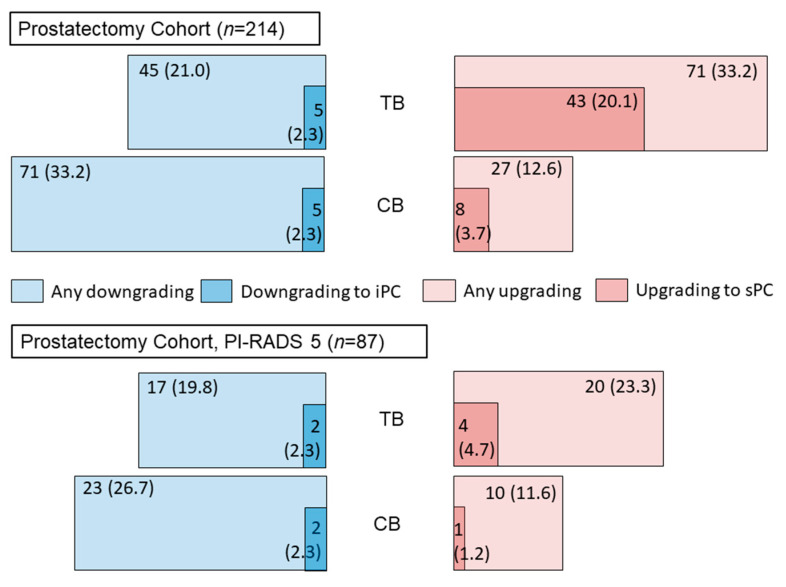
Reclassification rates of ISUP grade groups between prostate biopsy and a subsequent radical prostatectomy. For the whole prostatectomy cohort, combined biopsy mode (CB, MRI-targeted biopsy + synchronous systematic biopsy) reduced the upgrading to significant prostate cancer from 20.1% when considering MRI-targeted biopsy (TB) results only to 3.7%. For the subgroup of PI-RADS 5 men this effect turned neglectable (4.7% vs. 1.2%). iPC—indolent prostate cancer; sPC—significant prostate cancer; PI-RADS—Prostate Imaging Reporting and Data System; Length of bars is calculated by the respective percentages.

As a secondary endpoint, we analyzed the reclassification rates of ISUP grade groups in RP specimen with regard to the initial biopsy results (Figure 3). 214/1043 (20.5%) men of the total cohort underwent radical prostatectomy. Combined biopsy mode reduced the upgrading to sPC from 43/214 (20.1%) to 8/214 (3.7%) compared to TB only. When analyzing the subgroup of men with a PI-RADS 5 lesion, this difference decreased: 4/87 (4.7%) vs. 1/87 (1.2%).

## 4. Discussion

In PC diagnosis, SB is recommended to complement TB to address the limited sensitivity of TB alone [11]. SB may be beneficial for diagnosing additional sPC but also harmful in terms of additional iPC, morbidity, and resource expenditures. This study aimed to investigate selection criteria for men who should undergo SB within this clinically relevant trade-off.

### 4.1. Frequency of Upgrading to sPC by Additional Systematic Biopsy

In our retrospective cohort, additional SB upgraded the diagnosis to sPC in 98/1043 (9.4%) men. Previous studies similarly reported upgrading by adding SB but varied in the frequency of this event between 1.9–11.6% [8,16,17]. A recent meta-analysis found that omitting SB would even miss approximately 16% of sPC [5]. A reason for these deviations might originate from the various SB schemes applied and the different number of cores taken. The lowest frequency of upgrading was found in a multi-centre cohort where only eight SB cores were taken per patient [16]. In contrast to this, we applied the Ginsburg Scheme for SB and sampled a median of 26 systematic cores per patient. Acknowledging a targeting error as being the main reason for missing sPC by TB [4], the effect of additional SB might be mainly dependent on the surgeon’s experience. This reasoning is supported by the results of Sathianathen et al. who reported an upgrading of 11.6% by an additional 12-core SB in a cohort undergoing prostate biopsy by a group of surgeons without any experience in transperineal prostate biopsy before their study [8].

### 4.2. Predictors for Upgrading to sPC

When analysing predictors for the upgrading to sPC by additional SB, we found that men with PI-RADS 2-4 lesions were more likely to have their TB results upgraded by SB compared to PI-RADS 5 men. These results are in accordance with previous studies [7,18,19,20]. Ahdoot et al. proposed the patient’s PI-RADS score as a promising tool to select the optimal biopsy strategy in terms of omitting SB in men harbouring a PI-RADS 5 lesion [10].

Moreover, we found men with small lesions (<0.6 mL) and peripheral zone lesions more likely to have their diagnosis upgraded to significant disease by additional SB. The finding that smaller lesions are more probable to be missed by TB and thus lead to upgrading by an additional SB was previously described in a small cohort [9]. Altogether the option of an extended MRI-directed biopsy scheme is discussed in the current European Association of Urology guidelines to overcome the problem of targeting errors [11].

### 4.3. Stratification of Cancer Detection Rates by Patient’s PI-RADS Scores

Based on the finding that men harbouring PI-RADS lesions 2-4 were more likely to have their TB results upgraded by SB, we analyzed CDRs stratified by the patient’s respective PI-RADS score. In our cohort, we found men with PI-RADS 3 or PI-RADS 4 lesions more likely to be upgraded to sPC by an additional SB in 11.8% and 11.7% of cases, respectively. For PI-RADS 5 men, the added value of additional SB was reduced to 2.9% and without statistical significance compared to a TB-only strategy. Our results confirm those of Ahdoot and co-workers published for a cohort of 743 men who underwent TB plus synchronous 12 –core extended SB via the transrectal route: 7.5% upgrading to sPC in PI-RADS 3 men, 8.0% in PI-RADS 4 and 2.5% in PI-RADS 5 [10]. The slightly higher upgrading frequency in our study might originate from the application of the Ginsburg Scheme in our cohort, which conveys a higher number of SB cores taken.

### 4.4. Reclassification in Subsequent Radical Prostatectomy

Previous studies showed that CB is more predictive for a patient’s true pathological grade group compared to TB alone and thus reduces diagnostic uncertainty with a consecutive decrease of both over- and undertreatment [17]. In accordance with these previously published data, we found that adding SB to TB could reduce upgrading to sPC in a subsequent radical prostatectomy from 43/214 (20.1%) to 8/214 (3.7%). When analyzing the subgroup of men with a PI-RADS 5 lesion, this difference was negligible: 4/87 (4.7%) vs. 1/87 (1.2%). To our knowledge, this is the first subgroup analysis of a biopsy cohort for upgrading to sPC with wholemount specimen as the reference standard stratified by PI-RADS groups.

### 4.5. Limitations and Strengths of the Study

There were several limitations to the current study that must be acknowledged. The retrospective and single-center design limits the generalizability of our results. Moreover, our data originates from a cohort from a large academic center with a high level of expertise within the radiological and urological diagnostic prostate cancer pathways, which may not reflect the reality of care.

The study’s main strength is the evaluation of the additional effect of SB concerning reclassification rates in a subsequent radical prostatectomy, since this is fundamental to estimating the diagnostic uncertainty of the respective biopsy procedure. Moreover, this is the first study to evaluate the effect of additional SB stratified by PI-RADS groups in a large transperineal biopsy cohort.

### 4.6. Individualized Decision-Making

Our data suggest that the benefit of an additional SB in men with PI-RADS 5 lesions is limited since it only increases the rate of sPC diagnosis by 2.9%. This finding is confirmed when analyzing reclassification rates in a subsequent RP. Omitting SB in this group may result in fewer biopsy-related adverse events, a shorter operative time, lower procedure complexity, and a lower healthcare burden.

Nevertheless, the decision to perform only TB in PI-RADS 5 men must be taken individually. When considering focal therapy, CB provides information on the presence or absence of PC outside the MRI-lesion and thus is valuable for a precise surgical planning. Moreover, missing 2.9% of sPC in PI-RADS 5 men when omitting SB seems low but the risk threshold is ultimately subjective. Individual risk thresholds of both the physician and the patient must be considered when choosing the appropriate biopsy strategy.

## 5. Conclusions

Additional SB to complement TB conveys a trade-off between missing sPC on the one hand and the over diagnosis of iPC, and increased morbidity and resource expenditures on the other. We demonstrated that the benefit of an additional SB is small in men with a PI-RADS 5 lesion. This finding was confirmed when analyzing the diagnostic certainty of TB only versus TB+SB and comparing it with the reference standard of a subsequent radical prostatectomy. In conclusion, men with a PI-RADS 5 lesion may obviate additional SB.

## Figures and Tables

**Table 1:** Baseline Characteristics and Cancer Detection Rates. IQR—interquartile range; PSA—prostate specific antigen; PI-RADS—Prostate Imaging Reporting and Data System; ISUP—International Society of Urological Pathology.

Characteristic	All Men	Prostatectomy Cohort
Cases, n	1043	214
Age (years), median, IQR	67.0 (61.0–72.0)	67.0 (62.0–72.0)
Previous Negative Biopsy, n (%)	244 (23.4)	43 (20.1)
Active Surveillance, n (%)	141 (13.5)	29 (13.6)
PSA (ng/mL), median, IQR	8.8 (6.0–12.6)	9.3 (6.3–14.0)
Volume (mL), median, IQR	53.0 (38.5–75.0)	47.6 (37.0–63.3)
PI-RADS, n (%)		
n/a	54 (5.2)	8 (3.7)
1	0 (0.0)	0 (0)
2	43 (4.2)	6 (2.8)
3	170 (16.3)	17 (7.9)
4	530 (50.8)	97 (43.5)
5	246 (23.6)	86 (40.2)
Target Localization, n (%)		
Unilateral	595 (57.0)	116 (54.2)
Bilateral	448 (43.0)	98 (45.8)
Non-peripheral Zone	221 (21.2)	33 (15.4)
Peripheral Zone	444 (42.6)	103 (48.1)
Bi-zonal	378 (36.2)	78 (36.4)
Index Lesion Volume (mL), median, IQR	0.58 (0.32–1.14)	0.64 (0.30–1.52)
Number of Lesions, n (%)		
1	481 (46.1)	104 (48.6)
2	394 (37.8)	80 (37.4)
3	136 (13.0)	24 (11.2)
4 or more	32 (3.1)	6 (2.8)
Number of Cores, median, IQR		
Total	35 (31–40)	34 (30–39)
From Target	5 (3–7)	4 (4–7)
Systematic	31 (26–34)	30 (25–32)
Cancer Grading according to ISUP, n (%)		
No Cancer	394 (37.8)	n/a
1	128 (12.3)	8 (3.7)
2	174 (16.7)	71 (33.2)
3	142 (13.6)	82 (38.3)
4	162 (15.5)	26 (12.1)
5	43 (4.1)	27 (12.6)

**Table 2:** Univariate and multivariate logistic regression analysis for upgrading to significant prostate cancer (ISUP2–5) by systematic biopsy. Backward stepwise elimination was applied for variable selection in multivariable analysis. sPC—significant prostate cancer (ISUP > 2); ISUP—International Society of Urological Pathology; OR—odds ratio; CI—confidence interval; PSA—prostate specific antigen; PI-RADS—Prostate Imaging Reporting and Data System. * *p* < 0.05; ** *p* < 0.01.

Upgrading to sPC (ISUP2-5) by Systematic Biopsy				
	Univariate Analysis		Multivariate Analysis	
	OR (95% CI)	*p*	OR (95% CI)	*p*
Age, years	1.00 (0.97–1.03)	0.90		
Previous Negative Biopsy				
≥1 vs. none	0.94 (0.58–1.52)	0.78		
Active Surveillance				
Yes vs. No	0.86 (0.44–1.66)	0.65		
PSA level, ng/mL	0.99 (0.94–1.04)	0.72		
Prostate volume, mL	0.99 (0.98–1.00)	0.18	0.99 (0.98–1.00)	<0.03 *
PI-RADS Score				
PI-RADS 5	Ref.		Ref.	
PI-RADS 4 vs.	3.70 (1.66–8.28)	<0.01 **	4.62 (2.08–10.28)	<0.01 **
PI-RADS 3 vs.	4.43 (1.82–10.79)	<0.01 **	5.54 (2.26–13.57)	<0.01 **
PI-RADS 2 vs.	5.73 (1.80–18.26)	<0.01 **	7.37 (2.41–22.53)	<0.01 **
Target Localization				
Unilateral vs. Bilateral	0.99 (0.64–1.54)	0.97		
Non-peripheral Zone vs. Peripheral Zone	0.44 (0.23–0.85)	<0.01 **	0.42 (0.22–0.81)	<0.01 **
Bizonal vs. Peripheral Zone	0.55 (0.33–0.90)	0.02 *	0.70 (0.44–1.20)	0.14
Index Lesion Volume				
<0.6 mL vs. ≥ 0.6 mL	2.15 (1.38–3.34)	<0.01 **		
Number of lesions (n)				
1 vs. >1	0.99 (0.64–1.43)	0.97		
Number of Target Cores (n)	0.98 (0.89–1.07)	0.61		
Number of Systematic Cores (n)	1.03 (0.99–1.07)	0.11		

## Data Availability

The data presented in this study are available on request from the corresponding author.
